# Novel pulmonary abdominal normothermic regional perfusion circuit for simultaneous in-donor evaluation and preservation of lungs and abdominal organs in donation after circulatory death

**DOI:** 10.1007/s11748-025-02137-y

**Published:** 2025-03-16

**Authors:** Shin Tanaka, Masashi Umeda, Hiroyuki Ujike, Tsuyoshi Ryuko, Yasuaki Tomioka, Kentaroh Miyoshi, Mikio Okazaki, Seiichiro Sugimoto, Shinichi Toyooka

**Affiliations:** 1 Department of General Thoracic and Breast and Endocrinological Surgery, Dentistry and Pharmaceutical Sciences, Okayama University Graduate School of Medicine, 2-5-1, Shikata-cho, Kita-ku, Okayama, Japan; 2 Department of General Thoracic Surgery, Shimane University Graduate School of Medicine, Shimane, Japan

**Keywords:** Lung preservation, Donation after circulatory death, Abdominal normothermic regional perfusion

## Abstract

**Objective:**

To overcome limitations of traditional ex vivo lung perfusion (EVLP) for controlled donation after circulatory death (cDCD) lungs, this study aimed to evaluate a novel pulmonary abdominal normothermic regional perfusion (PANRP) technique, which we uniquely designed, for in situ assessment of lungs from cDCD donors.

**Methods:**

We modified the abdominal normothermic regional perfusion circuit for simultaneous lung and abdominal organ assessment using independent extracorporeal membrane oxygenation components. Blood was oxygenated via a membrane oxygenator and returned to the body, with pulmonary flow adjusted to maintain pressure < 25 mmHg. Femoral cannulation was performed, and the lungs were ventilated with standard settings. Organ function was assessed over 2 h using PaO2/FiO2, AST, ALT, BUN, and Cr measurements to monitor perfusion and oxygen delivery.

**Results:**

PANRP maintained stable lung function, with P/F ratios above 300, and preserved abdominal organ parameters, including stable AST, ALT, BUN, and Cr levels. Adequate urine output was observed, indicating normal renal function. Pulmonary artery pressure remained < 20 mmHg, and pulmonary vascular resistance was kept at 400 dyn・s/cm^5^, showing no signs of lung dysfunction or injury throughout the circuit.

**Conclusions:**

PANRP offers a promising alternative to traditional EVLP for cDCD lung evaluation, allowing in situ assessment of multiple organs simultaneously. This approach may overcome logistical and economic challenges associated with ex vivo techniques, enabling a more efficient evaluation process. Further studies are warranted to confirm its clinical applicability and impact on long-term outcomes.

## Introduction

Lung transplantation remains the leading treatment for end-stage lung disease, although the shortage of suitable donor organs continues [1, 2]. The use of lungs from controlled donors after circulatory death (cDCD) has contributed significantly to increasing the organ supply, and outcomes are equivalent to those obtained with donation after brain death (DBD) donors [3]. By 2018, DCD lungs represented 4.8% of all lung transplants, and further expansion of their use offers potential for continued growth in organ availability [4]. A significant distinction between cDCD and brain death organ transplantation lies in the primary cause of organ damage, which in the case of cDCD, is warm ischemic injury [5, 6]. In brain death, organs are procured immediately after aortic clamping, thus avoiding warm ischemic injury. The brief interval between aortic clamping and organ procurement allows for a reasonably reliable pre-explant assessment of organs in brain-dead donors. However, in cDCD, organ damage results from warm ischemic injury that occurs after cardiac arrest, which makes it impossible to directly apply the same pre-explant evaluation methods used for brain-dead donors. The duration and severity of warm ischemic time can vary considerably depending on the donor, and this damage cannot be reliably predicted before the donor's aortic clamping. As a result, distinct evaluation methods have been developed and implemented for assessing the extent of this injury across each organ individually [7–11].

In the assessment of liver and kidney donors, ex vivo evaluation systems have been employed. However, the current standard method increasingly adopted is abdominal normothermic regional perfusion (A-NRP) [8, 12]. A-NRP enables in situ evaluation of abdominal organs, allowing viable organs to be transported to the transplant facility while non-viable organs remain in the donor body. A-NRP is now routinely used in cDCDs in France, Italy, Spain, and the United Kingdom, and is being piloted in Belgium, the Netherlands, Norway, Switzerland and United States [13, 14].

In contrast, for lungs, in situ evaluation methods have not been developed, and ex vivo evaluation using ex vivo lung perfusion (EVLP) has become the globally accepted standard for assessing cDCD lungs [15]. Lungs are typically excised immediately after cardiac arrest and transported to the transplant facility for assessment using ex vivo methods, particularly when the warm ischemic period is prolonged. However, EVLP presents several limitations, including high costs [16], the need to transport the lungs to the transplant facility for evaluation, and the necessity of a complex process that involves cooling, warming, and re-cooling the lungs to preserve them.

To refine the standardized EVLP method, we developed a pulmonary ANRP (PANRP) technique that allows for in situ assessment of the lungs, similar to the evaluation process used for abdominal organs. PANRP facilitates in situ assessment of the lungs, aiming to alleviate the logistical and economic demands associated with ex vivo lung assessments, thereby offering a more effective and efficient alternative.

## Methods

### Overview of the circuit

**Fig. 1:**
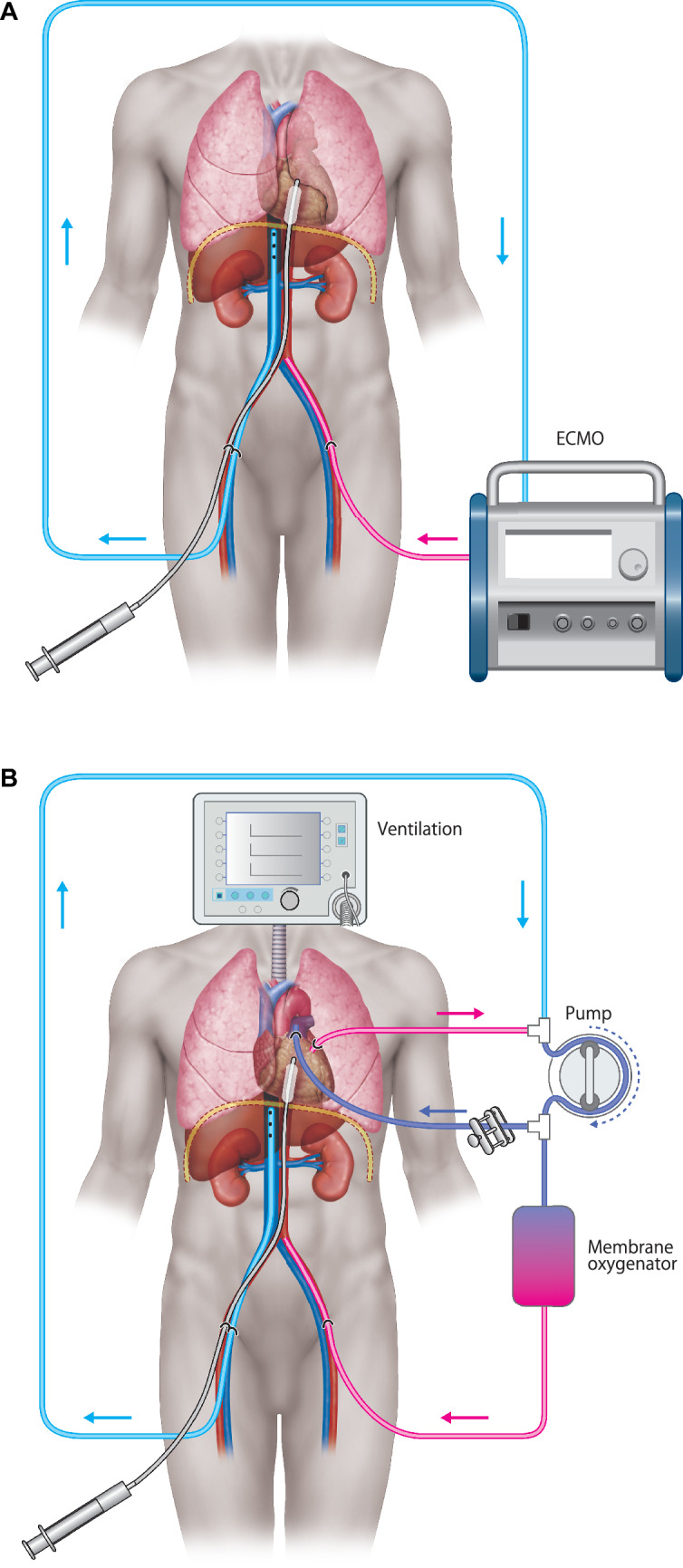

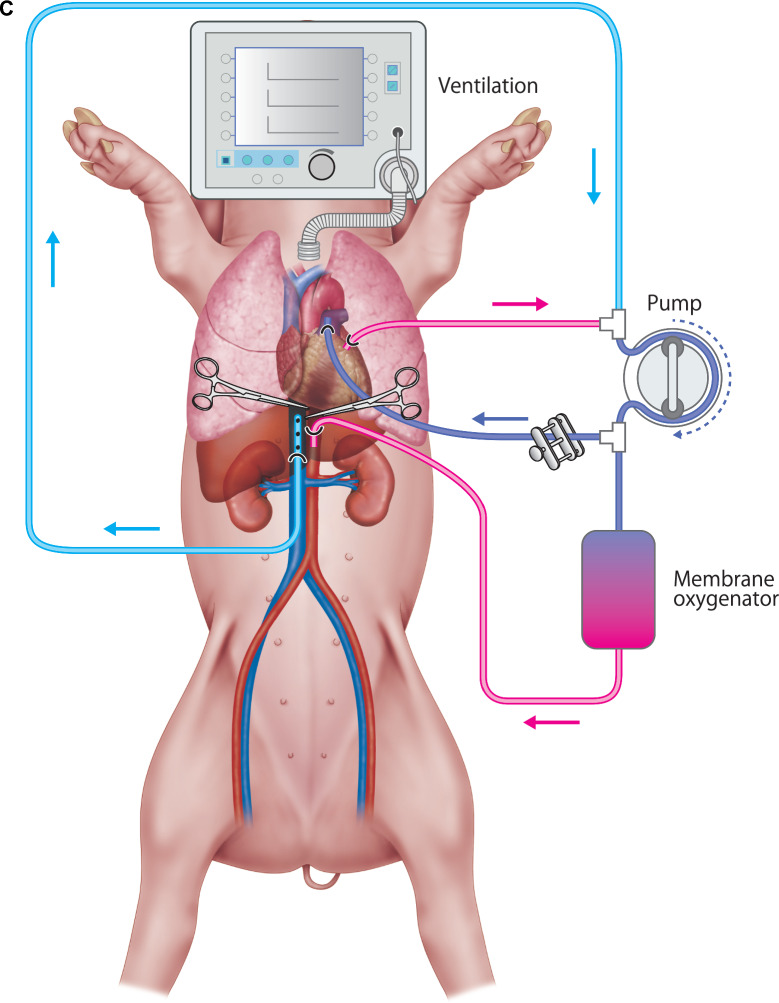
**A** Abdominal normothermic regional perfusion (ANRP). After cardiac arrest occurs in the donor, extracorporeal membrane oxygenation (ECMO) is performed to circulate blood flow to the abdomen to evaluate and protect the liver and kidney functions (Tanaka S. Effect on the donor lungs of using abdominal normothermic regional perfusion in controlled donation after circulatory death. Eur J Cardiothorac Surg. 2020; Nov 23: ezaa398). **B** Pulmonary abdominal normothermic regional perfusion (PANRP). By circulating a portion of the ANRP circuit to the lungs in the donor, they can be evaluated and preserved within the donor body. **C** PANRP circuit in a porcine experimental model. The diagram illustrates the experimental setup used to evaluate and preserve the lungs within the donor body by incorporating the PANRP circuit into the ANRP system, adapted specifically for preclinical research

This PANRP represents a modification of the existing A-NRP technique to facilitate the evaluation and preservation of both pulmonary and abdominal organs during the procedure. In A-NRP procedures, a extracorporeal membrane oxygenation (ECMO) system is utilized (Fig. 1A). However, in this circuit, we have specifically separated the ECMO components—using the pump and membrane oxygenator independently. Blood is drawn using the pump, then oxygenated via the membrane oxygenator, and subsequently returned to the donor’s body through the arterial line. A Y-connector is placed between the pump and membrane oxygenator to create a bypass to the lungs, allowing blood to bypass the pulmonary circulation. Blood is drained from the left atrium (LA) through another Y-connector and returned to the pump (Fig. 1B). While it may seem that the membrane oxygenator is unnecessary, as the lungs could oxygenate the blood, this is not feasible. The lungs in question are DCD lungs, which may be marginal or declined due to warm ischemic injury, so relying solely on pulmonary oxygenation is insufficient. Poor lung function could lead to decreased oxygen delivery to the abdominal organs, which is why the membrane oxygenator is included. The membrane oxygenator ensures that the abdominal organs receive adequate oxygen supply regardless of the condition of the lungs. Blood is circulated in this circuit for two hours, during which various organs are evaluated.

### Cannulation

**Fig. 2:**
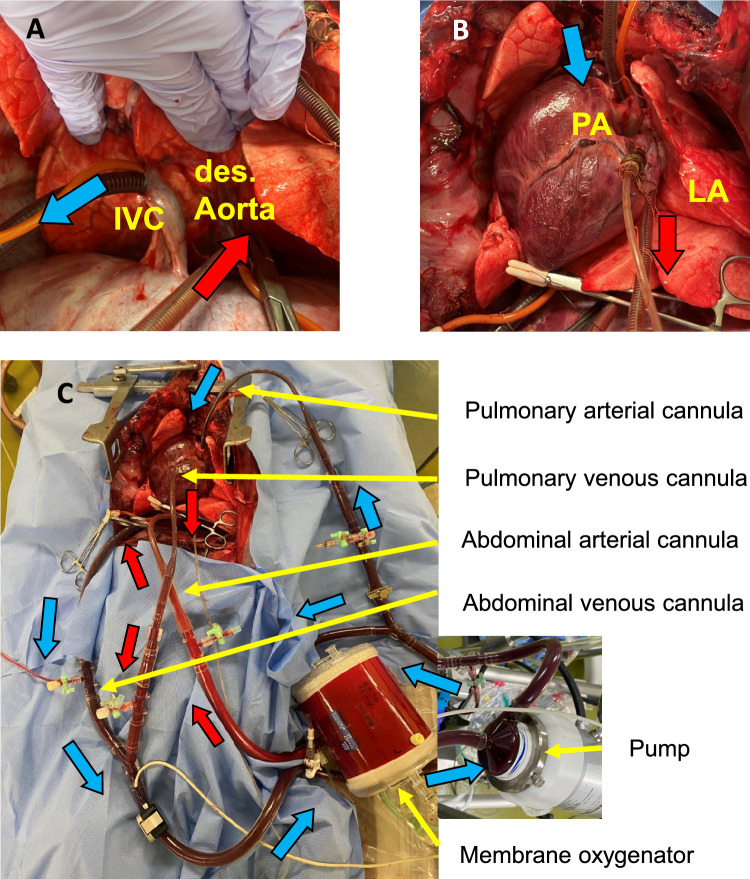
**A** Cannulation in pigs: The arterial cannula was inserted into the descending aorta, and the venous cannula was placed in the inferior vena cava after a median sternotomy and laparotomy. **B** Bypass circuit to the lungs: An 18-Fr arterial cannula was placed in the pulmonary artery, and a 22-Fr venous cannula was connected to the left atrium, establishing extracorporeal circulation.**C** Schematic of perfusion setup: The connection of the cannulas with the membrane oxygenator and the circulation circuit, simulating human clinical practice.

Male Landrace pigs were used as donor animals. Prior to organ retrieval, 10,000 units of heparin were administered to the donor. In human clinical practice, arterial and venous cannulation is performed through the femoral artery and vein before cardiac arrest. An 18-Fr arterial cannula would be inserted into the femoral artery, extending to the confluence of the right and left common iliac arteries. Similarly, an 18-Fr venous cannula would be inserted into the femoral vein, with the tip positioned at the level of the diaphragm, and both cannulas would be filled with heparinized perfusion solution. However, in pigs, the femoral vessels are too small to allow for percutaneous cannulation (Fig. 1C). Therefore, in this study, cardiac arrest was first induced by electrical cardioversion, followed by a median sternotomy and laparotomy to allow access to the intrathoracic descending aorta and inferior vena cava. After these vessels were exposed, an arterial cannula (normally inserted into the femoral artery in humans) was placed into the descending aorta at the diaphragm level, and a venous cannula (equivalent to femoral vein cannulation in humans) was placed into the inferior vena cava. Immediately after placing the arterial cannula into the descending aorta, a clamp was applied on the central (cardiac) side of the cannula (Fig. 2A). To establish a bypass circuit to the lungs, an 18-Fr arterial cannula was branched from the pump outlet and placed into the main pulmonary artery using a purse-string suture. A 22-Fr venous cannula was inserted into the left atrium and connected to the pump inlet (Fig. 2B). A warm ischemic time (WIT) of 30 min was set.

### Management during PANRP

The ECMO flow rate was set at 2–2.5 L/min, corresponding to approximately 60% of cardiac output. ECMO blood pressure was maintained at 60 mmHg or higher, as measured at the femoral artery, to ensure sufficient perfusion of the abdominal organs, and the ECMO temperature was controlled between 36.5 and 37.5 °C. The oxygen concentration in the membrane oxygenator was initially set at an FiO2 of 0.45–0.55, ensuring a PaO2 of at least 100 mmHg immediately after the membrane oxygenator in the circuit to guarantee adequate oxygen delivery to the abdominal organs, even in the case of compromised lung function.

The flow rate to the lungs was regulated using a pinch cock in the bypass vessel to maintain pulmonary artery pressure below 25 mmHg, resulting in a flow rate of 0.2–0.3 L/min. To confirm the absence of cerebral blood flow, pressure in the internal carotid artery was measured and consistently maintained at 0 mmHg throughout circuit operation. Albumin (25%) and hydroxyethyl starch were administered as needed to support systemic blood pressure and perfusion (Fig. 2C).

The lungs were ventilated with standard settings (tidal volume 9–10 mL/kg, FiO2 0.5, positive end-expiratory pressure [PEEP] 5 cm H2O, respiratory rate [RR] 6 breaths per minute).

### Assessment of lung, liver, and kidney functions

During the two-hour lung evaluation, pulmonary function is monitored by tracking the PaO2/FiO2 (P/F) ratio, mean pulmonary pressure, pulmonary vascular resistance, and blood flow to the donor lungs, ensuring all parameters remain within the desired ranges. The liver and kidney functions are measured over time using aspartate transaminase (AST), alanine transaminase (ALT), blood urea nitrogen (BUN), and creatinine (Cr), which remain constant until the end of the 2-h circuit.

### Study approval

All animals received humane care in compliance with the Principles of Laboratory Animal Care (formulated by the National Society for Medical Research) and the Guide for the Care and Use of Laboratory Animals (prepared by the Institute of Laboratory Animal Resources and published by the National Institutes of Health; NIH publication no. 86–23 revised in 1996). The study protocol was approved by the Animal Care and Use Committee of Okayama University (protocol #**OKU- 2,023,579**).

## Results

Three large animal experiments were performed.

### Pulmonary functional assessment

**Fig. 3:**
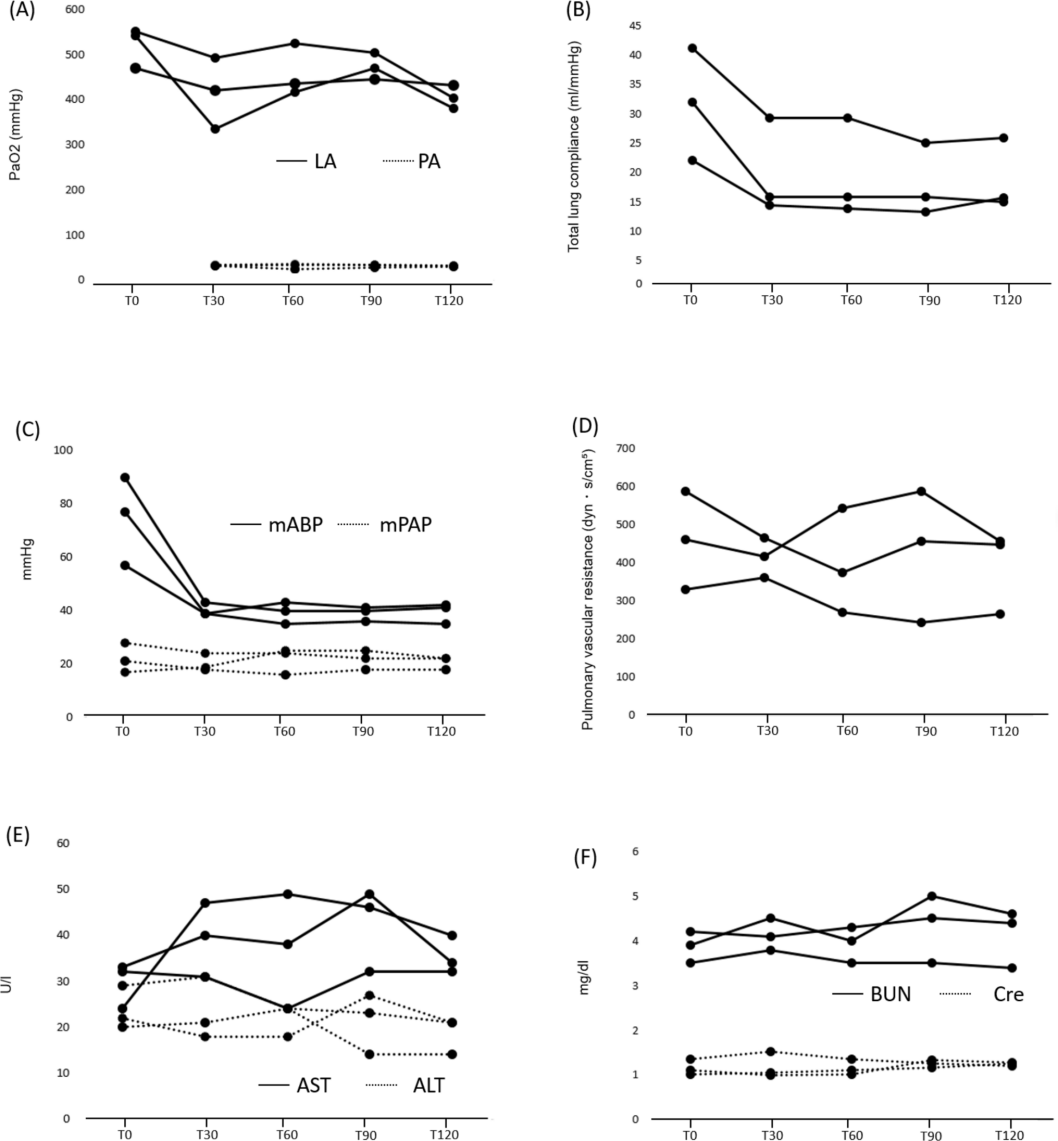
Organ assessment parameters over time during PANRP. **A** Partial pressure of oxygen in arterial blood (PaO_2_)/fraction of inspired oxygen (FiO_2_) ratio in the left atrium (LA) and pulmonary artery (PA). **B** Mean arterial blood pressure (mABP) and mean pulmonary artery pressure (mPAP). **C** Total lung compliance. **D** Pulmonary vascular resistance. **E** Aspartate aminotransferase (AST) and alanine aminotransferase (ALT). **F** Blood urea nitrogen (BUN) and creatinine (Cre)

**Fig. 4:**
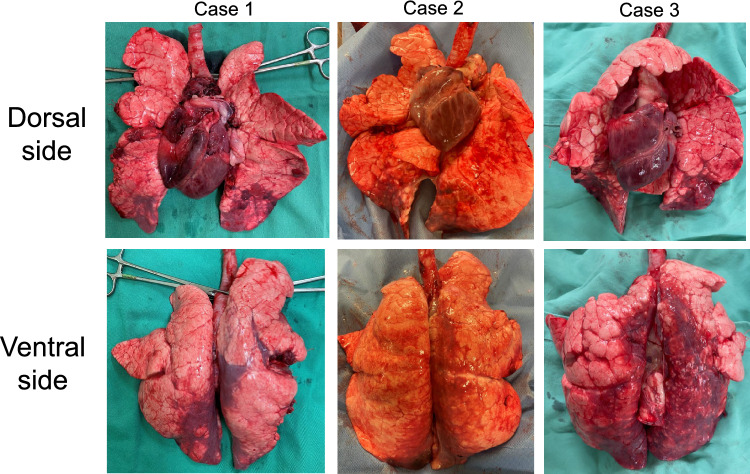
At the end of the procedure, the excised lungs from both cases exhibited small areas of atelectasis in both lower lobes. However, no signs of redness, additional atelectasis, edema, or other localized abnormalities were observed in three cases

Throughout the circuit, the P/F ratio in the LA was consistently maintained above 300, while the partial pressure of oxygen in the pulmonary artery remained stable. The oxygen extraction (ΔPaO2), representing the difference between the PaO2 in the LA and the pulmonary artery, was also kept above 300 (Fig. 3A). Tidal volume was set at 250 mL and was maintained without no significant changes in airway pressure or lung compliance (Fig. 3B). The total pump flow rate was 2.8 L, with 0.2 L allocated to thoracic perfusion. Mean pulmonary artery pressure remained consistently below 30 mmHg (Fig. 3C), and Pulmonary vascular resistance was maintained at 400 dyn・s/cm^5^ (Fig. 3D). In both cases, the lungs demonstrated stable function throughout the two-hour circuit, with no signs of redness, atelectasis, edema, or localized abnormalities in appearance at the end of the procedure (Fig. 4).

### Abdominal organ functional assessment

Clinical parameters for abdominal organs, including AST, ALT, BUN, and Cr, were monitored similarly to those employed in A-NRP. Throughout the circuit, no abnormal values were detected, and all parameters consistently remained within normal ranges (Fig. 3E, F). During the PANRP circuit, sufficient urine output was observed in both cases, with volumes exceeding 200 mL over the two-hour period. Perfusion flow to the abdomen was maintained between 2 and 2.5 L, while the perfusion pressure for abdominal circulation was sustained above 60 mmHg.

## Discussion

In this study, we have introduced a novel circuit for in situ evaluation of cDCD lung donors, leading the way globally in this innovative approach. This circuit not only facilitates the assessment and protection of the donor lungs but also allows for simultaneous evaluation and protection of the abdominal organs. We believe this circuit will make a significant contribution to the broader adoption of cDCD transplantation.

For cDCD involving the lungs, organs are typically removed immediately after the confirmation of the donor’s death. The primary cause of damage to organs in donation after circulatory death (DCD) is warm ischemic injury. After the donor is withdrawn from mechanical ventilation, the systolic blood pressure gradually decreases. It is generally accepted that warm ischemic injury begins when the systolic blood pressure drops below 60 mmHg. The interval from when the systolic blood pressure falls below 60 mmHg to the initiation of cold perfusion of the organ is considered the warm ischemic time (WIT). The acceptable WIT varies by organ: for the liver and kidneys, it is around 30 min, while for the lungs, due to their ability to retain oxygen in the alveoli, it is generally considered to be up to 60 min [17]. Lung damage increases with the duration of the WIT, and most of the injury occurs during the cardiac arrest phase. As a result, lung function assessment has traditionally been performed ex vivo after organ removal [18]. Although ex vivo evaluation systems exist for abdominal organs, in Europe, A-NRP has been widely adopted for cDCD involving abdominal organs. This approach has improved graft utilization and outcomes in DCD transplants [19–21]. While in situ evaluation methods have been established for abdominal organs, they had not been applied to lung assessment. Therefore, we developed a new PANRP circuit based on the abdominal organ evaluation system. This circuit allows for the in situ evaluation of donor lungs within the donor body.

Traditionally, lung evaluation has been conducted ex vivo using costly perfusion solutions after organ removal, as the presence of cytokines that promote inflammatory responses poses a risk to lung tissue. While this ex vivo system was initially developed for DCD lungs, its usefulness has also been demonstrated in brain-dead donors, and it has become part of the standard lung transplant process [18]. However, there are several limitations associated with this approach. First, the cost is significant. The implementation of EVLP incurs substantial additional costs, approximately $20,000 to $50,000 perprocedure, and requires a specialized team to operate [16, 22]. Moreover, not all lungs assessed with EVLP are suitable for transplantation, with utilization rates varying significantly from 34.0% to 96.9% [23], which can further drive up the overall cost per transplant. A recent UK study estimated an incremental cost of approximately $116,000 per quality-adjusted life-year gained [16]. Additionally, in ex vivo lung evaluation systems, the lungs are first re-warmed for assessment and then re-cooled for preservation until transplantation. This process involves two periods of cold ischemia, which may cause additional damage to the donor lungs.

In contrast, abdominal organs have benefited from in situ evaluation methods that avoid these issues. While there was concern that inflammatory cytokines in the circuit might damage transplant organs, a recent study comparing A-NRP and super-rapid recovery (SRR) for controlled donation after circulatory death (cDCD) showed that abdominal organs perfused with A-NRP for two hours had superior outcomes. A-NRP was linked to fewer biliary complications (8% vs. 31%) and lower graft loss (12% vs. 24%), demonstrating that it preserves organ viability and improves graft function and survival [24]. Moreover, the ability to maintain good pulmonary function while using the PANRP circuit suggests that in situ evaluation of cDCD could be a feasible and potentially effective approach, offering benefits from a medical resource standpoint. This circuit overcomes the disadvantages of ex vivo evaluation, including cost, recipient burden, and the risk of temperature-induced damage to donor lungs. To further scientifically validate the utility of this circuit, future studies should focus on the outcomes of organ transplantation, pathological evaluations of the lungs post-transplantation, and blood cytokine concentration measurements following the use of PANRP. Additionally, it is necessary to analyze the post-transplant outcomes of lungs evaluated with PANRP to confirm its effectiveness. Comparing the inflammatory profiles of PANRP and existing EVLP systems will also be crucial for addressing concerns regarding inflammatory cytokine responses during perfusion. If non-inferiority of PANRP to EVLP can be demonstrated, it will provide strong evidence to support the clinical utility and accuracy of PANRP in evaluating DCD lungs.

For clinical implementation of the PANRP circuit, ensuring ethical compliance is essential. A critical requirement is the ability to demonstrate complete cessation of cerebral blood flow during the circuit's operation. In this study, cerebral blood flow was monitored by measuring carotid artery pressure throughout the procedure, which was consistently maintained at 0 mmHg, confirming the absence of cerebral perfusion. This ensured that the circuit operated without compromising ethical standards. In future clinical applications, bispectral index monitoring could complement this approach to provide additional confirmation of the cessation of brain activity, aligning with ethical requirements for DCD. From a technical standpoint, the PANRP circuit can be constructed using existing equipment, similar to the ANRP circuit, facilitating a smooth transition to clinical application. The procedure for simultaneous evaluation of lungs and abdominal organs with PANRP aligns closely with standard ANRP workflows. In ANRP protocols, after the donor’s death is confirmed, the thoracic and abdominal cavities are opened, the lungs are promptly explanted, and the abdominal organs are evaluated in situ by monitoring changes in organ discoloration and other perfusion parameters. This established surgical workflow can be seamlessly adapted for PANRP. By enabling in-donor evaluation of DCD lungs, the PANRP circuit is expected to reduce the incidence of primary graft dysfunction (PGD), a major acute complication following lung transplantation. Furthermore, this approach holds promise for improving long-term pulmonary function after transplantation, emphasizing the potential clinical value of integrating PANRP into DCD transplantation practices.

A limitation of this method is that it is difficult to use in conjunction with the retrieval of the donor heart in cDCD, particularly when thoracic normothermic regional perfusion (TANRP) is employed, as TANRP is specifically designed for the retrieval and evaluation of hearts from cDCD donors. TANRP plays a critical role in cDCD heart transplantation, facilitating in situ cardiac evaluation [25]. Another approach for cDCD hearts, ex vivo extraction and evaluation using an organ care system, further complicates the cannulation process designed for PANRP due to the absence of the heart at the initiation of the circuit. While it may be possible to effectively drain and collect blood from an open LA, this aspect requires further refinement in circuit design. Therefore, the PANRP circuit is most suitable for cases where cardiac retrieval is not intended.

## Conclusion

The study concludes that the PANRP circuit offers a feasible and potentially effective method for in situ evaluation of cDCD donor lungs, addressing some limitations of ex vivo approaches such as high costs and logistical burdens. By maintaining lung function during the evaluation and reducing the risk of temperature-induced damage, PANRP presents a promising alternative. However, further studies are necessary to confirm its efficacy, particularly through post-transplant outcomes and pathological assessments of the lungs following transplantation.

## Data Availability

Data are available upon request from the authors.
